# Coupled and decoupled legumes and cereals in prehistoric northern and southern China

**DOI:** 10.3389/fpls.2022.1013480

**Published:** 2022-10-07

**Authors:** Keyang He, Xiaoshan Yu, Caiming Shen, Houyuan Lu

**Affiliations:** ^1^ Key Laboratory of Cenozoic Geology and Environment, Institute of Geology and Geophysics, Chinese Academy of Sciences, Beijing, China; ^2^ State Key Laboratory of Tibetan Plateau Earth System, Resources and Environment, Institute of Tibetan Plateau Research, Chinese Academy of Sciences, Beijing, China; ^3^ Yunnan Key Laboratory of Plateau Geographical Processes and Environmental Changes, Faculty of Geography, Yunnan Normal University, Kunming, China; ^4^ Innovation Academy for Earth Science, Chinese Academy of Sciences, Beijing, China; ^5^ College of Earth and Planetary Sciences, University of Chinese Academy of Sciences, Beijing, China

**Keywords:** soybeans, fish, millet and rice, agricultural systems, late Yangshao period

## Abstract

Legumes and cereals, which provide different nutrients, are cultivated as coupled crops in most centers of plant domestication worldwide. However, as the only legume domesticated in China, the spatio-temporal distribution of soybeans and its status in the millet- and rice-based agricultural system of the Neolithic and Bronze Ages remains elusive. Here, archaeobotanical evidence of soybeans (n=254), millet (n=462), rice (n=482), and zooarchaeological evidence of fish (n=138) were synthesized to elucidate the phenomenon of coupled or decoupled cereals and legumes in prehistoric China. During the Neolithic and Bronze Ages, soybeans was mostly confined to northern China and rarely found in southern China, serving as a companion to millet. In contrast, fish remains have been widely found in southern China, indicating a continuous reliance on fish as a staple food besides rice. Thus, an antipodal pattern of millet-soybeans and rice-fish agricultural systems may have been established in northern and southern China since the late Yangshao period (6000–5000 cal BP) respectively. These two agricultural systems were not only complementary in terms of diet, but they also exhibited positive interactions and feedback in the coculture system. Consequently, these two systems enabled the sustainable intensification of agriculture and served as the basis for the emergence of complex societies and early states in the Yellow and Yangtze Rivers.

## Introduction

**Figure 1:**
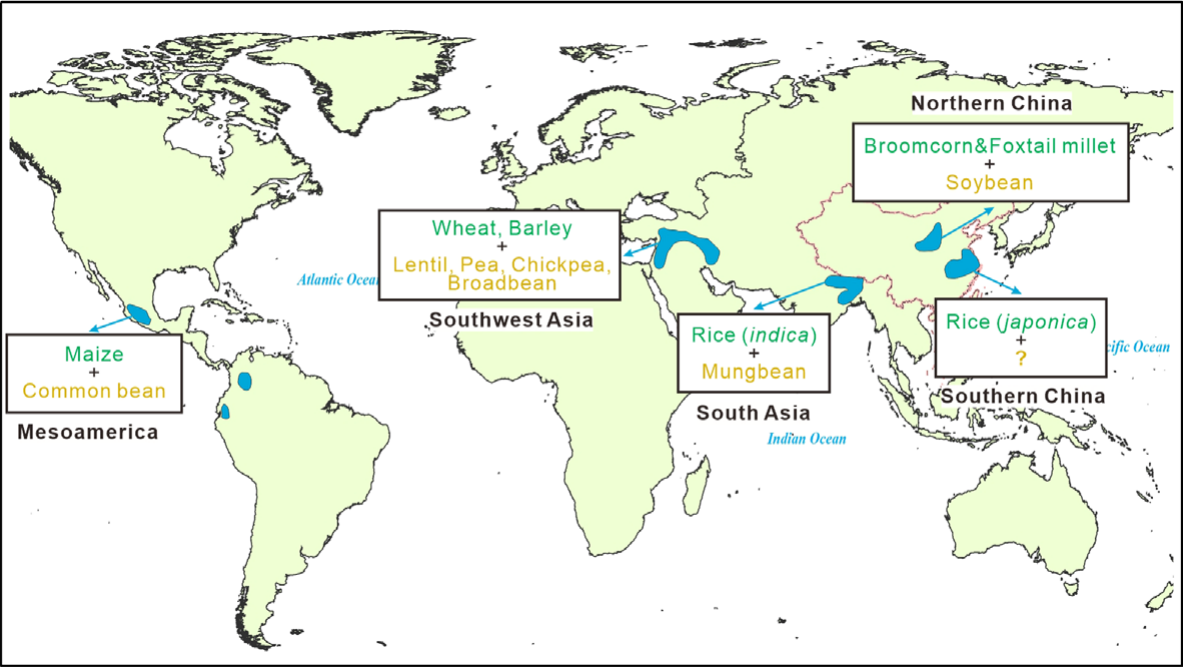
Staple cereals and companion legumes in the centers of plant domestication (Larson et al., 2014).

Optimal foraging theory in anthropological studies suggests that energy constraints and nutrient requirements are vital to the origin and evolution of agriculture (Smith et al., 1983) . As essential dietary sources of protein and starch, legumes and cereals are nutrient complements (Zohary et al., 2012) and thus have been cultivated as coupled crops in most centers of origin of agriculture worldwide (**Figure 1**) (Purugganan and Fuller, 2009; Larson et al., 2014), even if pre-domestication cultivation may occur broadly in different regions within these centers (Willcox, 2013; Arranz-Otaegui et al., 2016). Maize (*Zea mays*) in Mesoamerica was accompanied by the common bean (*Phaseolus vulgaris*) (Piperno, 2011), wheat (*Triticum aestivum*) and barley (*Hordeum vulgare*) in southwest Asia had a series of companion legumes, including lentil (*Lens culinaris*), pea (*Pisum sativum*), chickpea (*Cicer arietinum*), and broadbean (*Vicia faba*) (Zeder, 2011), and rice (*Oryza sativa* subsp. *indica*) in south Asia was associated with the mungbean (*Vigna radiata*) (Fuller, 2011). However, soybeans (*Glycine max*) seem to be merely associated with broomcorn (*Panicum miliaceum*) and foxtail (*Setaria italica*) millets in northern China, while no companion legumes to rice (*Oryza sativa* subsp. *japonica*) have been discovered in southern China.

Despite China being generally regarded as the geographical origin of cultivated soybeans (Carter Jr. et al., 2004), the exact location of soybean domestication is still in dispute. Due to the widespread distribution of its wild relatives–wild soybeans (*Glycine soja*) in far eastern Russia, China, Korea, and Japan, a variety of regions have been proposed as candidate sources (Zhao and Gai, 2004) based on the genomic analysis of modern soybeans and archaeological evidence of charred soybeans (Sedivy et al., 2017), such as northeastern China, the middle and lower Yellow River in northern China (Li et al., 2008; Han et al., 2016), the Yangtze River in southern China (Guo et al., 2010), and multiple domestications in East Asia including the Yellow River valley in China, Korea, and Japan (Lee et al., 2011). Nevertheless, the dispute can largely be attributed to the lack of comprehensive research on the spatio-temporal distribution of cultivated and wild soybeans recovered in prehistoric China.

Furthermore, the role of soybeans as a staple crop and their relationship to other cereal crops has rarely been discussed. Soybeans contains approximately 40% protein (Zong et al., 2017), ranking the highest protein content among all cultivated crops, and thus may serve as an important source of protein in addition to livestock during the transition from a mobile hunter/gatherer society to a sedentary agricultural society (Zhou et al., 2011; Yuan et al., 2020). However, archaeobotanical evidence has suggested that soybeans were merely added to the cropping patterns in northern China and was known as one of the “five grains” contributing to the formation of ancient civilization in the Central Plains (Zhao, 2011; Liu et al., 2015). In contrast, fish, which consists of approximately 20% protein, was intensively exploited in the coastal or wetland environments in southern China (Yuan et al., 2008) and has been argued to co-evolve with wet rice cultivation (Nakajima et al., 2019). Thus, whether fish might have replaced soybeans as an important source of protein in prehistoric southern China still needs to be verified by archaeological evidence.

As an important source of plant protein, soybeans played a significant role in supplementing starchy foods like millet and rice in the dietary structure of ancient humans. To investigate the spatio-temporal distribution of soybeans and its status in the millet- and rice-based agricultural system in prehistoric China (He et al., 2017), we synthesized both archaeobotanical evidence of soybeans, millets, and rice across China together with zooarchaeological evidence of fish in southern China during the Neolithic and Bronze Age (**Supplementary Materials**) and attempted to elucidate the phenomenon of coupled or decoupled cereals and legumes in northern and southern China.

## Material and methods

**Figure 2:**
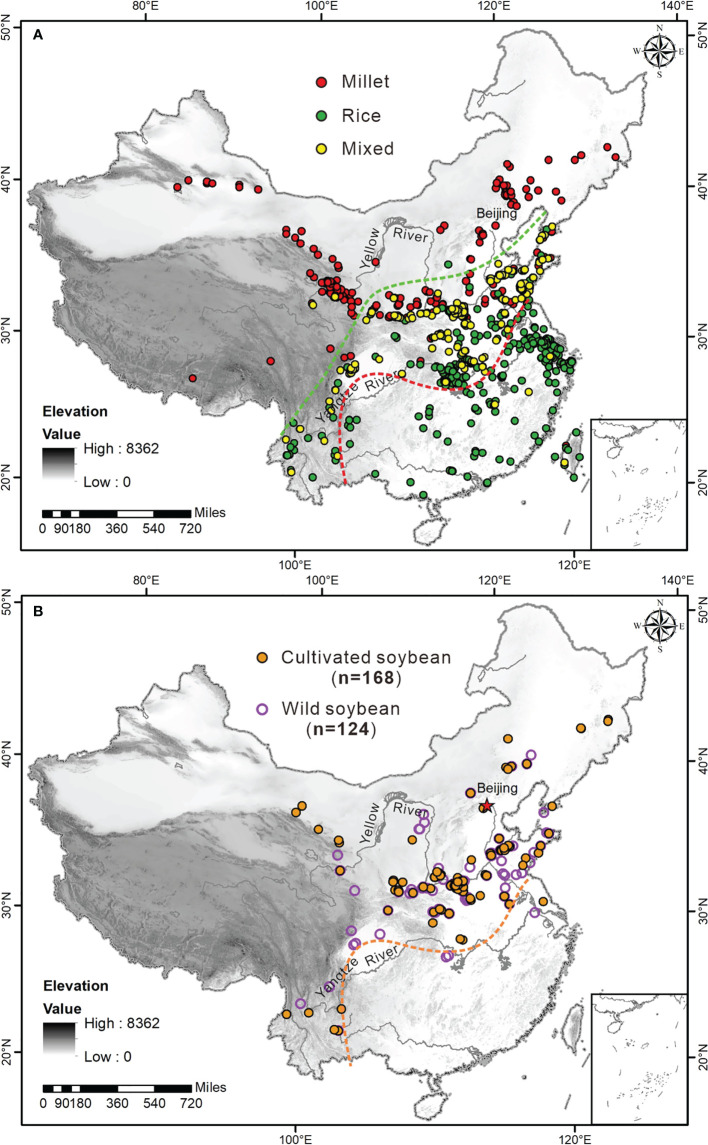
Distribution of staple cereals (millet and rice) **(A)** (He et al., 2017) and companion legumes (soybeans) **(B)** in the Neolithic and Bronze Age across China. The red, green and yellow solid circulars in **(A)** indicate sites of millet, rice and mixed farming, respectively. The orange solid and purple hollow circulars in **(B)** denote sites of cultivated and wild soybeans respectively.

Most of the archaeobotanical data used here were flotation results systematically collected from a series of published research papers, reports, and dissertations. A total of 254 sites with soybeans excavated during different periods were compiled (**Figure 2**), including 168 sites of cultivated soybeans and 124 sites of wild soybeans. The flotation results were recounted with uniform standards that excluded pieces of crop seeds, and different parts of the crop seeds were added up. Except for sites without exact numbers published, the maximum number of cultivated soybeans and wild soybeans excavated was 1057 in the Yuanqiao site (Fuller and Zhang, 2007) and 1116 in the Jiaojia site (Wu, 2018) respectively. Data on millets and rice were obtained from (He et al., 2017), which included 462 sites of millet and 482 sites of rice.

**Figure 3:**
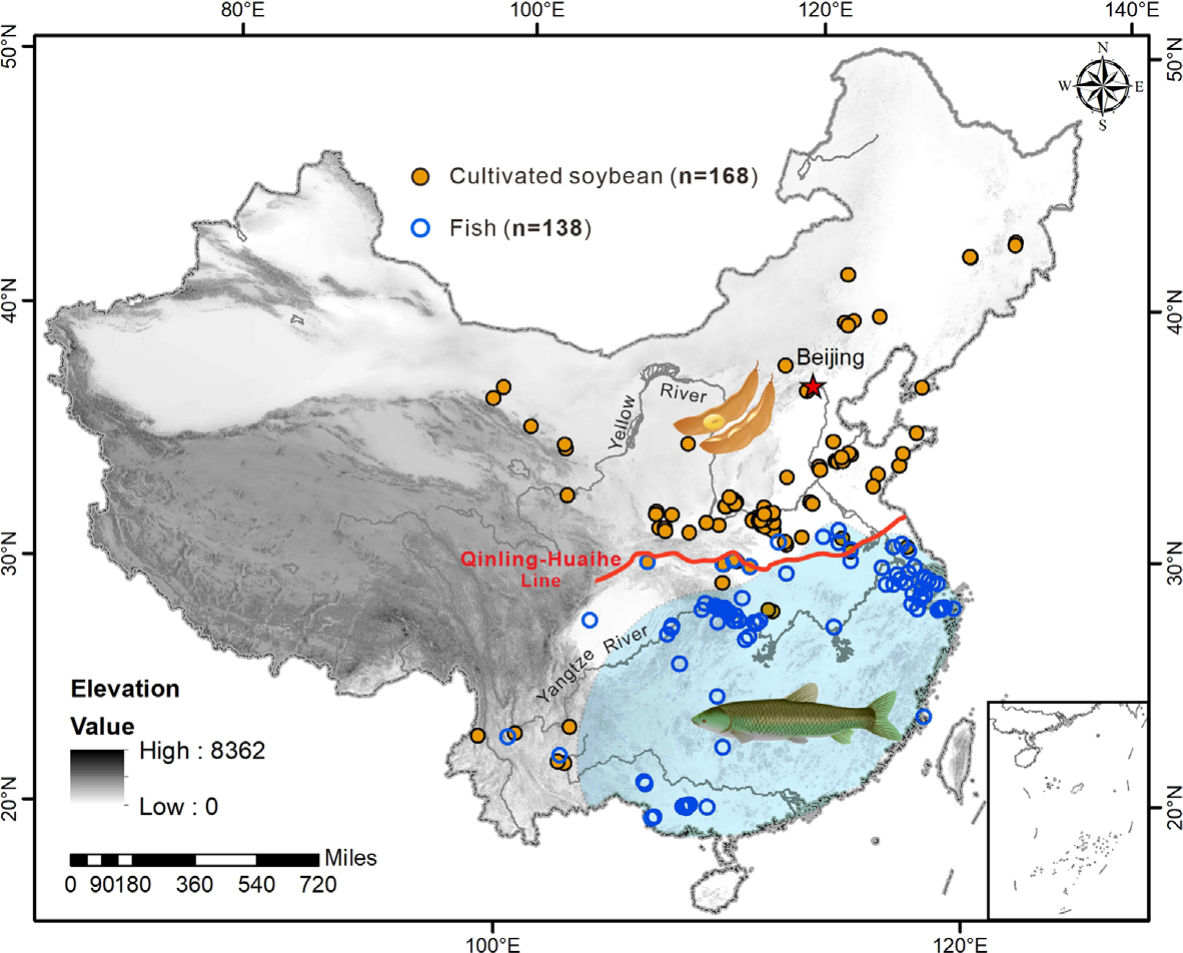
Contrastive patterns of soybeans and fish in the Neolithic and Bronze Age across China. The orange solid and blue hollow circulars in denote sites of cultivated soybeans and fishes, respectively.

The zooarchaeological data used here were obtained by a review of the relevant literature (Li and Luo, 2014; Mo, 2016; Zhu et al., 2020; Liang and Hu, 2022). A total of 138 sites with fish remains excavated during different periods were compiled (**Figure 3**), including 89 sites from the Yangtze River (20, 35, and 32 sites in the upper, middle, and lower reaches, respectively), 32 sites from the Pearl River, 15 sites from the Huai River, and 2 sites from Southeast China.

All the sites during the Neolithic and Bronze Ages were classified into five periods, i.e., Peiligang period (8000–7000 cal BP), Early Yangshao period (7000–6000 cal BP), Late Yangshao period (6000–5000 cal BP), Longshan period (5000–4000 cal BP), and Bronze Age (4000–2221 cal BP) (The Institute of Archaeology and China Academy of Social Sciences, 2010). Subsequently, the sites of soybeans, wild soybeans, and fish remains were plotted based on the five cultural periods using ArcMap 10.6.

## Results and discussion

### Coincidence between soybeans and millet

Based on the previous investigations on the distribution of millet, rice, and mixed farming sites (He et al., 2017), the millet agricultural system was mostly confined to northern China except for the Chengdu Plain-Yungui Plateau and Hanshui Valley in the upper and middle Yangtze River respectively (**Figure 2A**). Noticeably, the distribution of soybeans exhibits a striking coincidence to those of millet (**Figure 2B**), with the northeastern-most site is Damudantun (129°18′E, 44°15′N, ~3000 cal BP) in Ningan, Heilongjiang Province and the southwestern-most site is Shilinggang (98°52′E, 25°39′N, ~2700 cal BP) in Lushui, Yunnan Province. Thus, the soybeans may have undergone coevolution with millet considering that soybeans are able to fix atmospheric nitrogen and improve soil fertility while millet absorbs soil nitrogen (Zohary et al., 2012).

### Preliminary rice–fish coculture system

In contrast to the dense distribution of soybeans in northern China, soybeans were rarely found in southern China except for a few sites in the middle Yangtze River (**Figure 3**), e.g., soybeans were present at the Qujialing site (n=6) and wild soybeans at the Bashidang site (n=27). Instead, fish had been exploited intensively near various water bodies like rivers, lakes, wetlands, and paddy fields in the Yangtze River region (**Figure 3**), such as the Zhongba (Flad, 2005) and Tianluoshan sites (Zhang, 2018) where thousands of fish remains were excavated. The major fish species exploited in the Yangtze River were from the family of Cyprinidae, including black carp (*Mylopharyngodon piceus*), grass carp (*Ctenopharyngodon idella*), and crucian carp (*Carassius carassius*) (Li and Luo, 2014; Mo, 2016). Besides, the fish remains retrieved from the middle Yangtze River were entirely composed of freshwater species, while those in the lower Yangtze River were supplemented by several coastal species, such as the flathead grey mullet (*Mugil cephalus*) (Mo, 2016).

In terms of meat acquisition strategies, fishes were clearly an important component of diets in the Yangtze River throughout the Neolithic Age, whereas they seemed to be less significant in the Yellow River (Yuan et al., 2008). A detailed analysis of predominant fish in the Tianluoshan site confirms that fishing played a significant role in the subsistence economy and mostly occurred in a rather concentrated area of the wetlands (Zhang, 2018). Specially, rice paddy was also discovered in the Tianluoshan site through the fire and flood management of coastal marsh (Zheng et al., 2009). Thus, a preliminary rice–fish coculture system may have been established during the Hemudu culture (7000–5000 cal BP) based on the positive interactions between fish and rice, where the fishes reduced the number of rice pests, and rice helped moderate the water environment for fish. This system has been practiced by farmers for over 1300 years in Qingtian from the southern Zhejiang province, China (Xie et al., 2011).

In addition to fishes, shellfishes were also regarded as an important source of protein, which was mostly confined to the coastal regions, especially the Liaodong and Shandong peninsula to the northeast and Fujian and Liangguang region to the southeast with hundreds of shell middens existed during 7000–5000 cal BP (Zhang and Hung, 2016). However, shellfishing played different roles in the subsistence of these two regions. In the Liaodong and Shandong peninsula, shellfishing may have been embedded in the agricultural system of millet and soybean. By contrast, shellfishing along with hunting and gathering was the predominant subsistence in the Fujian and Liangguang region before the introduction of rice agriculture around 5000 cal BP (Yang et al., 2018).

### Antipodal patterns of soybeans and fish

**Figure 4:**
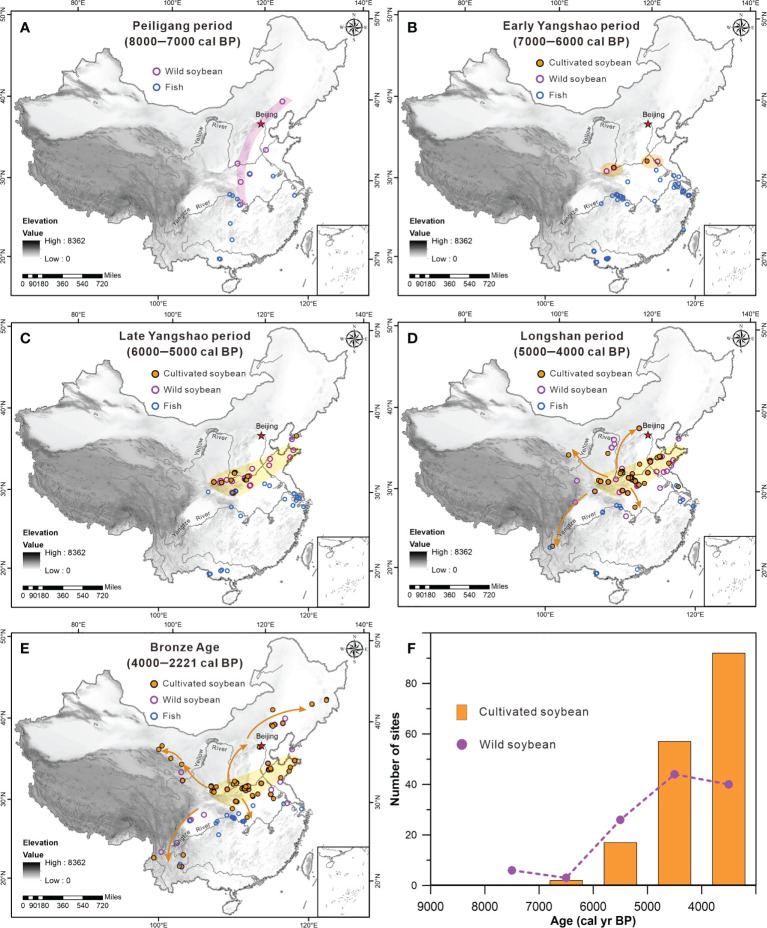
Diachronic evolution of soybeans and fish in the Neolithic and Bronze Age across China. The orange solid and purple hollow circulars denote sites of cultivated and wild soybeans respectively. The light-colored shades denote the core region of soybean, and the orange arrows indicate the spread routes of soybean.

Given that soybeans and fish served as an important source of protein for ancient humans in northern and southern China, antipodal patterns of a millet-soybean and rice-fish agricultural system may have formed in the Neolithic and Bronze Ages. During the Peiligang period (8000-7000 cal BP), only wild soybeans was discovered along the marginal mountains of the Loess Plateau and Inner Mongolian Plateau (**Figure 4A**) (Liu et al., 2009), while fishes had been exploited in the middle and lower Yangtze River and Pearl River. Subsequently, cultivated soybeans emerged in the middle and lower Yellow River during the Yangshao period (7000–5000 cal BP) (**Figures 4B, C**), with the earliest dates in the Dongyang (~6000 cal BP) (Zhao, 2019) and Helou (~6200 cal BP) (Zhong et al., 2021) sites. Consistent with new archeological evidence, whole-genome analysis suggests that domestication of soybeans likely occurred 6000–9000 years ago (Kim et al., 2010) in the middle and lower Yellow River (Zhang and Singh, 2020). Alternatively, fishing activity in the middle and lower Yangtze River increased greatly during this period.

During the Longshan period (5000–4000 cal BP) and Bronze Age (4000–2221 cal BP), the soybeans began to spread in four directions (**Figures 4D, E**): northwestward to the Ganqing region, southwestward to the Yungui Plateau, north-northeastward to the Yanbei and Liaoxi regions, and southward to the Hanshui valley. The number of soybean sites thus increased dramatically (**Figure 4F**). Meanwhile, the seed size of soybeans increased notably and can be divided into two size groups (Lee et al., 2011; Wu et al., 2013). However, the seed size was influenced by a variety of factors (Zhao and Yang, 2017) such as regional difference and charring shrinkages, which indicates that the later stage of domestication syndrome may have been delayed by ~2000 years (Fuller, 2007; Chen et al., 2017). In contrast, ancient humans in the middle and lower Yangzi River barely used soybeans and continued to rely on an abundant supply of fishing throughout the Neolithic into Bronze Age.

In summary, antipodal patterns of a millet-soybeans agricultural system in northern China and rice-fish agricultural system in southern China may have been established since the late Yangshao period (6000–5000 cal BP), which sustained the nutritional requirement of both starch and protein. To a certain extent, the cultivation and domestication of soybeans in the Yellow River may compensate for the possible lack of protein due to the transition from hunting wild animals to the rearing of livestock. However, the relative abundance of diverse animals and fish species in the Yangtze River may promote ancient humans to continue fishing and hunting rather than reliance on soybeans domestication (Yuan et al., 2008), and ultimately lead to the decoupled cereal and legumes in southern China.

## Conclusion

In most centers of origin of agriculture in the world, legumes and cereals have been cultivated as coupled crops except for southern China. Soybeans, generally considered to be domesticated in the middle and lower Yellow River, was mostly confined to northern China and served as a companion to millets. In contrast, fish had been intensively exploited in southern China and rarely recovered from the Yellow River. Thus, since the late Yangshao period (6000–5000 cal BP), an antipodal pattern of millet-soybeans and rice-fish agricultural systems may have been established in northern and southern China, respectively. On one hand, these two systems are dietary complementation of starch and protein, providing balanced requirements of energy and nutrient; on the other hand, the systems exhibit agronomic compensation that soybeans fix atmospheric nitrogen and improves the soil fertility while millet uses soil nitrogen up, and fish reduce rice pests and rice favors fish by moderating the water environment. Positive interactions in these two systems enabled sustainable intensification in agriculture and provide the basis for the emergence of complex societies and early states in the Yellow and Yangtze Rivers.

## Data availability statement

The original contributions presented in the study are included in the article/**Supplementary Material**. Further inquiries can be directed to the corresponding authors.

## Author contributions

HL designed the research. XY and KH assisted the analysis of archaeobotanical and zooarchaeological data. XY and CS contributed to the discussion sections. KH compiled the figures. KH and HL wrote the manuscript. All authors contributed to the article and approved the submitted version.

## Funding

This research was supported by the National Natural Science Foundation of China (Nos. 41830322, T2192954, 41902187 , and 42177437), and China Postdoctoral Science Foundation (Nos. 2020M670444).

## Acknowledgments

We sincerely thank Xue Yan, Xiaoqu Zheng, Yuqian Wang and YM for their assistance with data collection. We would like to thank Editage (www.editage.cn) for English language editing.

## Conflict of interest

The authors declare that the research was conducted in the absence of any commercial or financial relationships that could be construed as a potential conflict of interest.

The handling editor YG declared a shared affiliation with the authors KH, XY, HL at the time of review.

## Publisher’s note

All claims expressed in this article are solely those of the authors and do not necessarily represent those of their affiliated organizations, or those of the publisher, the editors and the reviewers. Any product that may be evaluated in this article, or claim that may be made by its manufacturer, is not guaranteed or endorsed by the publisher.
